# FOSTERING MULTIDISCIPLINARY SOLUTIONS IN AGING: THE RESEARCH CENTERS COLLABORATIVE NETWORK

**DOI:** 10.1093/geroni/igac059.424

**Published:** 2022-12-20

**Authors:** Stephen Kritchevsky, Odette van der Willik

## Abstract

The NIA supports the Research Centers Collaborative Network (RCCN) to build collaborations between scientists from the 6 NIA-sponsored center programs: Alzheimer's Disease Research Centers, Centers on the Demography and Economics of Aging, Claude D. Pepper Older Americans Independence Centers, Nathan Shock Centers of Excellence in the Basic Biology of Aging, Resource Centers for Minority Aging Research, and Roybal Centers for Translational Research on Aging. RCCN's premise is that researchers from different disciplines are most likely to collaborate when they are addressing common problems. To provide a forum for collaborative exchange, the RCCN has convened 6 workshops on topics that cross-cut the concerns of the various NIA center programs ranging from sustaining behavior change in older adults to measuring biologic age. After each Workshop the RCCN awards pilot funds related to the theme. This symposium will present key learnings from the workshops and present work of four RCCN pilot teams from the third and fourth workshops which focused on resilience and reserve, and life course perspectives on aging. Dr. Ramos will discuss how measurement of psychological resilience may predict physical resilience in older patients with lung cancer, while Dr. Reid will discuss positive affect as a source of resilience for older adults with chronic pain. Dr. White will discuss association between perceived discrimination trajectories and multimorbidity burden among middle‐aged and older black adults, while Dr. Brooks will discuss development of a resilience index in chronic musculoskeletal pain. Dr. Kritchevsky will also summarize lessons learned across the first 6 workshops.

